# Persistence of *Burkholderia thailandensis* E264 in lung tissue after a single binge alcohol episode

**DOI:** 10.1371/journal.pone.0218147

**Published:** 2019-12-10

**Authors:** Victor M. Jimenez, Erik W. Settles, Bart J. Currie, Paul S. Keim, Fernando P. Monroy

**Affiliations:** 1 Department of Biological Sciences, Northern Arizona University, Flagstaff, AZ, United States of America; 2 Pathogen & Microbiome Institute (PMI), Northern Arizona University, Flagstaff, AZ, United States of America; 3 Menzies School of Health Research, Charles Darwin University, Darwin, Australia; University of Toledo College of Medicine and Life Sciences, UNITED STATES

## Abstract

**Background:**

Binge drinking, an increasingly common form of alcohol use disorder, is associated with substantial morbidity and mortality; yet, its effects on the immune system’s ability to defend against infectious agents are poorly understood. *Burkholderia pseudomallei*, the causative agent of melioidosis can occur in healthy humans, yet binge alcohol intoxication is increasingly being recognized as a major risk factor. Although our previous studies demonstrated that binge alcohol exposure increased *B*. *pseudomallei* near-neighbor virulence *in vivo* and increased paracellular diffusion and intracellular invasion, no experimental studies have examined the extent to which bacterial and alcohol dosage play a role in disease progression. In addition, the temporal effects of a single binge alcohol dose prior to infection has not been examined *in vivo*.

**Principal findings:**

In this study, we used *B*. *thailandensis* E264 a close genetic relative of *B*. *pseudomallei*, as useful BSL-2 model system. Eight-week-old female C57BL/6 mice were utilized in three distinct animal models to address the effects of 1) bacterial dosage, 2) alcohol dosage, and 3) the temporal effects, of a single binge alcohol episode. Alcohol was administered comparable to human binge drinking (≤ 4.4 g/kg) or PBS intraperitoneally before a non-lethal intranasal infection. Bacterial colonization of lung and spleen was increased in mice administered alcohol even after bacterial dose was decreased 10-fold. Lung and not spleen tissue were colonized even after alcohol dosage was decreased 20 times below the U.S legal limit. Temporally, a single binge alcohol episode affected lung bacterial colonization for more than 24 h after alcohol was no longer detected in the blood. Pulmonary and splenic cytokine expression (TNF-α, GM-CSF) remained suppressed, while IL-12/p40 increased in mice administered alcohol 6 or 24 h prior to infection. Increased lung and not intestinal bacterial invasion was observed in human and murine non-phagocytic epithelial cells exposed to 0.2% v/v alcohol *in vitro*.

**Conclusions:**

Our results indicate that the effects of a single binge alcohol episode are tissue specific. A single binge alcohol intoxication event increases bacterial colonization in mouse lung tissue even after very low BACs and decreases the dose required to colonize the lungs with less virulent *B*. *thailandensis*. Additionally, the temporal effects of binge alcohol alters lung and spleen cytokine expression for at least 24 h after alcohol is detected in the blood. Delayed recovery in lung and not spleen tissue may provide a means for *B*. *pseudomallei* and near-neighbors to successfully colonize lung tissue through increased intracellular invasion of non-phagocytic cells in patients with hazardous alcohol intake.

## Introduction

Alcohol-use disorders (AUDs) have long been allied to increased vulnerability to lung infections. Observations by the first surgeon general of the United States indicated that individuals with an affinity for alcohol had a higher incidence of pneumonia and tuberculosis [1]. Compared to non-binge drinkers, patients with a history of alcohol abuse are twice as likely to develop alcohol-induced lung injury and immune dysfunction that contributes to a higher risk for developing respiratory infections, leading to increased morbidity and mortality [2]. The emerging tropical disease melioidosis is characterized by pneumonia in half of all reported cases, with reported mortality rates as high as 50% [3]. *Burkholderia pseudomallei* is the causative agent of melioidosis and is a Tier 1 select agent. The genus *Burkholderia* contains over 40 species and includes less-pathogenic *Burkholderia thailandensis*, which coexists with *B*. *pseudomallei* in the soil in melioidosis-endemic regions but has also been identified sporadically in the midwestern United States [4, 5]. The presence of one or more risk factors have been observed in 80% of confirmed melioidosis cases, with nearly 40% of Australian cases having hazardous alcohol use as a risk factor [6]. Worldwide, up to 30% of patients with AUDs are disparately affected by *Streptococcus pneumoniae*, the most common causative agent in bacterial pneumonia. [7].

Additionally, hazardous alcohol consumption has been shown to alter the initial host-pathogen interactions during infections caused by *Mycobacterium avium*, *Escherichia coli*, *Streptococcus pneumoniae*, *Klebsiella pneumoniae*, *Staphylococcus aureus*, and *B*. *thailandensis*. [8, 9, 10, 11]. Moreover, the immunologic effects of alcohol differ depending on the pattern of alcohol consumption; binge (4 or 5 drinks for women and men, respectively at a single session) and chronic (7 or 14 drinks/ wk for women and men, respectively) alcohol consumption alters innate immune cells and worsens mortality after infection in humans and in animal models [12, 13]. Most studies indicate binge alcohol consumption inhibits signaling pathways downstream of toll-like receptors (TLRs), including the NF-κB pathway in murine and human cells; whereas, chronic alcoholism produces amplified signaling in the same pathway [14]. Binge alcohol consumption results in a reduction of proinflammatory cytokines in response to TLR stimulation or even when TLR expression is not altered [15]. Studies in both human and animal models indicate that chronic alcohol ingestion impairs the capacity of alveolar macrophages to phagocytose and clear bacteria due to desensitization of the cilia in the upper airway [16]. The lungs may be exceptionally susceptible to alcohol damage due to their delicate architecture and high cellular exposure to vapor phase alcohol during breathing [17]. However it remains unclear how a minimum infectious dose, a low single binge alcohol dose, and the temporal effects of binge alcohol intoxication alter the susceptibility of tissue colonization during a *Burkholderia* infection. More specifically, the way in which first time alcohol use from a binge-like dose affects the development of pneumonic melioidosis.

In our previous studies, we found that a single binge alcohol episode alters alveolar macrophage phagocytosis and increases intracellular survival of *B*. *thailandensis in vitro* [18]. Additionally, our lab has shown that after a single binge alcohol episode, infectivity with less-pathogenic *B*. *thailandensis* can increase 24 h post intranasal infection, while diffusing into the blood stream, compared to no detectable bacteria in major organs when alcohol is not administered [11]. From these findings we concluded that a single exposure of binge alcohol intoxication increased the infectivity and dissemination of less pathogenic *B*. *thailandensis* E264 out of the lung and into other vital organs by suppressing the initial host immune response and facilitating bacterial movement through paracellular space and intracellular invasion of epithelial and endothelial cells. However, the effects of varied bacterial–alcohol doses on lung colonization or the temporal effects of binge alcohol intoxication during a *B*. *thailandensis* infection have not been determined. In this study we designed three independent binge alcohol intoxication mouse models to investigate: 1) the effects of bacterial dosage during a single binge alcohol episode on lung and spleen colonization of less pathogenic *B*. *thailandensis E264*, 2) the effects of alcohol dosage during a single moderate infection of *B*. *thailandensis* on lung and spleen colonization, 3) the temporal effects of a single binge alcohol episode on *B*. *thailandensis* lung and spleen colonization. Our results indicate that lung tissue is unable to clear a low *B*. *thailandensis* infection after a single binge alcohol episode or with very low alcohol exposure, while lung tissue remains more susceptible to infection and the immunologic effects from alcohol that is administered 24 h prior to infection.

## Materials and methods

### Bacterial growth and culture conditions

For each study, frozen stock cultures (*B*. *thailandensis E264*) were inoculated into Luria Bertani broth (LB) and incubated overnight at 37°C in an orbital shaker incubator (200 rpm) (New Brunswick C25, Edison, NJ, USA). Bacteria were diluted 1:10 and grown to late-logarithmic phase measured by optical density at OD_600_ absorbance in a spectrophotometer (Eppendorf Bio Photometer AG2233, Hamburg, Germany). Bacteria were collected in 1mL by centrifugation and resuspended in 1mL with pre-warmed Dulbecco’s Phosphate-Buffered Saline (PBS) at an actual concentration of 10^5^ cfu/25μL depending on the particular assay. Actual numbers of viable bacteria were determined by standard plate counts of the bacterial suspensions on LB agar plates. The Pathogen & Microbiome Institute (PMI), Northern Arizona University, USA, kindly provided *B*. *thailandensis* E264. All animal experiments were performed with 6 mice per group and at least two independent experiments were completed with similar results.

### Animals

This study was carried out in strict accordance with the recommendation in the Guide for the Care and Use of Laboratory Animals of the National Institutes of Health. The protocol was approved, and animal care use was conducted in accordance with the Institutional Animal Care and Use Committee (IACUC) according to the policies and procedures of Northern Arizona University, (approval number 16–006). Animals were euthanized under isoflurane anesthesia, and all efforts were made to minimize suffering. Female 8-10-week-old C57BL/6 mice (Jackson Laboratory) with a body weight of 17–21 g were maintained on a standard laboratory chow ad libitum and were housed in a controlled environment with a 12-h light/dark cycle. After receipt, the mice were allowed to acclimate and recover from shipping stress for 5 days in our university laboratory animal facility, which is evaluated by the American Association for Accreditation of Laboratory Animal Care (AAALAC) for adherence to federal regulations. These mice were negative for common mouse pathogens during the period of this study.

### Binge alcohol animal models

Binge alcohol intoxication was induced by intraperitoneal (IP) injection of 20% alcohol in sterile tissue-culture grade water (Sigma Chemical Co., St. Louis, MO) maintained at room temperature. Mice had not been primed previously with alcohol consumption. Control mice received an equal volume of PBS IP. The following three independent binge alcohol animal models were designed and implemented:

***B*. *thailandensis* dosage and binge alcohol intoxication.** Each mouse was administered a single dose of alcohol (4.4 g/kg) that produced a peak BAC of ~ 0.254%. This BAC represents the higher end of the range observed in humans, but it is not particularly rare and has been reported as a common BAC in human binge drinkers in a number of studies [19]. Equally, mice eliminate alcohol from their systems more rapidly than humans. Producing biologically equivalent effects of alcohol in mice, as in human binge drinkers, requires a higher dosage in mice. Briefly, viable *B*. *thailandensis* at non-lethal doses of 10^5^, 10^4^, 10^3^, or 10^2^ CFU or PBS were administered in 25 μl intranasally 0.5 h after IP injection of alcohol or PBS.

**Alcohol dosage, blood alcohol concentration and a single exposure to *B*. *thailandensis* E264.** Each mouse was administered a single dose of alcohol (4.4, 3, 2, or 1 g/kg) that produced a peak BAC of ~ 0.254, 0.152, 0.0265, or 0.00397%. These BACs represent a range of the higher end of binge drinking observed in humans, and the legal limits defined by the United States (0.08%) or Australia (0.05%). This BAC range produces biologically equivalent effects of alcohol in mice, as in human alcohol drinkers, when comparing BAC and associated estimated number of standard drinks for men and women (Table 1). Briefly, viable *B*. *thailandensis* (10^5^ CFU) were administered in 25 μl intranasally 0.5 h after IP injection of alcohol or PBS.

**Table 1:** Mouse blood alcohol concentrations (BAC) and associated estimated number of standard drinks for men and women. Approximate blood alcohol in 1–2 hours for men and women (140–180 lb.).

Estimated Number of Standard Drinks			
BAC (%)	mg/dl	Men	Women
0.254	254.2	9–11	8–9
0.152	152.3	6–7	4–6
0.0265	26.5	2–5	1–3
0.00397	3.97	≤ 1	≤ 1

One standard drink is based on 1.5 oz. of 80 proof liquor (40%), 12 oz. beer (4.5%), or 5 oz. wine (12%).

Source: National Highway Traffic Safety Administration, USA.

#### Temporal effects of binge alcohol intoxication and a single exposure to *B*. *thailandensis* E264

Each mouse was administered a single dose of alcohol (4.4 g/kg) that produced a peak BAC of ~ 0.254%. Briefly, viable *B*. *thailandensis* (10^5^ CFU) were administered in 25 μl intranasally 0.5, 3, 6, or 24 h after IP injection of alcohol or PBS. Peak BAC was achieved at 0.5 h post alcohol administration with a decline until no alcohol is detected in the blood at 8 h post alcohol administration, as described in Jimenez et al. [11].

Inoculums were administered into each nostril under isoflurane anesthesia. Mice were monitored to observe differences in exploratory and motor control characteristics, in addition to physical health. Mice were weighed pre-infection and post-infection prior to euthanasia. Mice were subsequently euthanized at 24 h after the intranasal injection. At this time point, depending on the experimental protocol, aortic blood was taken for bacterial counts or lung and spleen tissues were removed and processed to quantify bacterial load and as an indicator of dissemination. Tissue homogenates were utilized to quantify cytokine expression. Mice were divided into four groups, and no bacteria was cultured from non-infected mice. At least two independent animal experiments per model were run with similar results.

### Blood alcohol concentration and bacteriology of blood and tissues

Binge alcohol was administered as a single dose of a 20% (weight/volume) alcohol solution in sterile water by IP injection during the light cycle. Alcohol was injected in mice by using a 27-guage X 0.5-inches (0.4mm X 13mm) needle. All animals were deprived of food and water for 1 h before administration of alcohol but retained free access to food and water post alcohol administration. Blood samples were collected prior to infection in 20 μL heparinized capillary tubes and transferred to 1.5-mL vials that were septum-sealed and stored at 4° C until analyzed.

Blood alcohol concentration measurements and quantification of bacteria in blood and tissues were conducted as described in Jimenez et al. [11]. Bacteriology in blood and tissue assays were run in at least triplicate and at least two independent experiments were performed with similar results.

### GM-CSF, TNF-α, IL-12/p40 and IL-10 tissue cytokine measurements

Lung and spleen tissue homogenates collected at 24 PI were utilized to quantify GM-CSF, TNF-α, IL-12/p40, and IL-10. Samples were measured using ELISA Ready-SET-Go kits (Affymetrix-eBioscience, San Diego, USA) with procedures supplied by the manufacturer. The minimum detectable levels of GM-CSF, TNF-α, IL-12/p40 and IL-10 were 4, 8, 4, and <13 pg/mL, respectively. In brief, culture plates were coated with goat anti-mouse GM-CSF, TNF-α, IL-12/p40, or IL-10 capture antibody and were incubated overnight at 4° C. After the plates were washed, wells were blocked and incubated for 1 h at room temperature. After several washes, respective standards and samples were added to each well, and were incubated overnight at 4° C for maximal sensitivity. After several more washes biotinylated anti-mouse detection antibody was added to each well, and the plate was incubated at room temperature for 1 h. Streptavidin-horseradish peroxidase then was added, and the plate was incubated for 30 min at room temperature. After a final wash, peroxidase substrate TMB solution was added and incubated at room temperature in the dark for 15 min. Adding 3 M sulfuric acid to each well stopped the reaction. Color development in each well was determined spectrophotometrically at 450 nm (Synergy HT, BioTek, Winooski, USA). GM-CSF, TNF-α, IL-12/p40, or IL-10 results are expressed as pg/mL. Cytokine assays were run in six assay replicates and repeated independently at least twice with similar results.

### Binge alcohol and non-phagocytic cells: Live *Burkholderia* intracellular invasion assays

*B*. *thailandensis* cell invasions with and without alcohol exposure was measured using Murine lung (Eph4, LET-1), intestinal (Mode K), or human lung (A549) epithelial cells. Cell monolayers were grown with DMEM F12 medium (Gibco, Life Technologies) supplemented with 10% fetal bovine serum, 2 mM L-glutamine, 10 mM HEPES, 0.1 mM non-essential amino acids, 1.5 g/l sodium bicarbonate, 50 U/ml penicillin, and 50 mg/ml streptomycin. Cells were incubated at 37^o^ C and 5.5% CO_2_ prior to and after confluency. Cell monolayers were incubated in DMEM F12 media supplemented with 0% or 0.2% (v/v) alcohol for 3 h prior to infection or at the time of infection. Low evaporative cell culture plates and a compensating system were employed as described by [20]. In addition, alcohol- and control non-supplemented media changes were used to ensure consistent alcohol concentrations. Alcohol concentration was selected based on ≥ 90% cell viability utilizing the Trypan blue exclusion cell viability test. Alcohol concentration was also consistent with average mouse BAC.

*B*. *thailandensis* was grown overnight in sterile LB media. Prior to co-culturing conditions, the bacteria were diluted to late logarithmic growth, centrifuged, and the pellet was washed twice in fresh non-antibiotic DMEM F12 media. Cell monolayers were then co-cultured with *B*. *thailandensis* at a MOI of 1:10 for 3 h at 37^o^ C, 5.5% CO_2_ to allow intracellular invasion to occur. After 3 h, extracellular bacteria were removed by washing cells with PBS and replacing culture media supplemented with 250 μg/ml of kanamycin for 1 h. Thereafter, the cell monolayers were incubated (37^o^ C) in media containing 50 μg/ml kanamycin for 1 h for a total of 2 h antibiotic treatment to completely kill any residual extracellular and attached bacteria. Following an additional PBS wash, intracellular bacteria were released after cell monolayers were lysed with PBS containing 0.1% Triton X-100 (total assay incubation time was 5 hours after initial monolayer exposure to bacteria). Viable intracellular bacteria were quantified by plating serial dilutions of the lysate, and average CFU determined. Bacterial intracellular invasion assays were performed in experimental and assay triplicates and replicated independently at least twice.

### Statistical analysis

The data analysis was completed using Prism 5.0 software (Graph Pad, 5.04, San Diego, CA). Assay replicate independence was determined by a one-way or two-way ANOVA with Bonferroni multiple comparisons, and Student’s *t*-test. Additional statistics were performed using R, and non-parametric, unequal variances. A P value of less than 0.05 was considered significant.

## Results

### *B*. *thailandensis* persists in lung tissue when alcohol is administered 0.5 h prior to infection, regardless of low infectious dose

**Fig 1:**
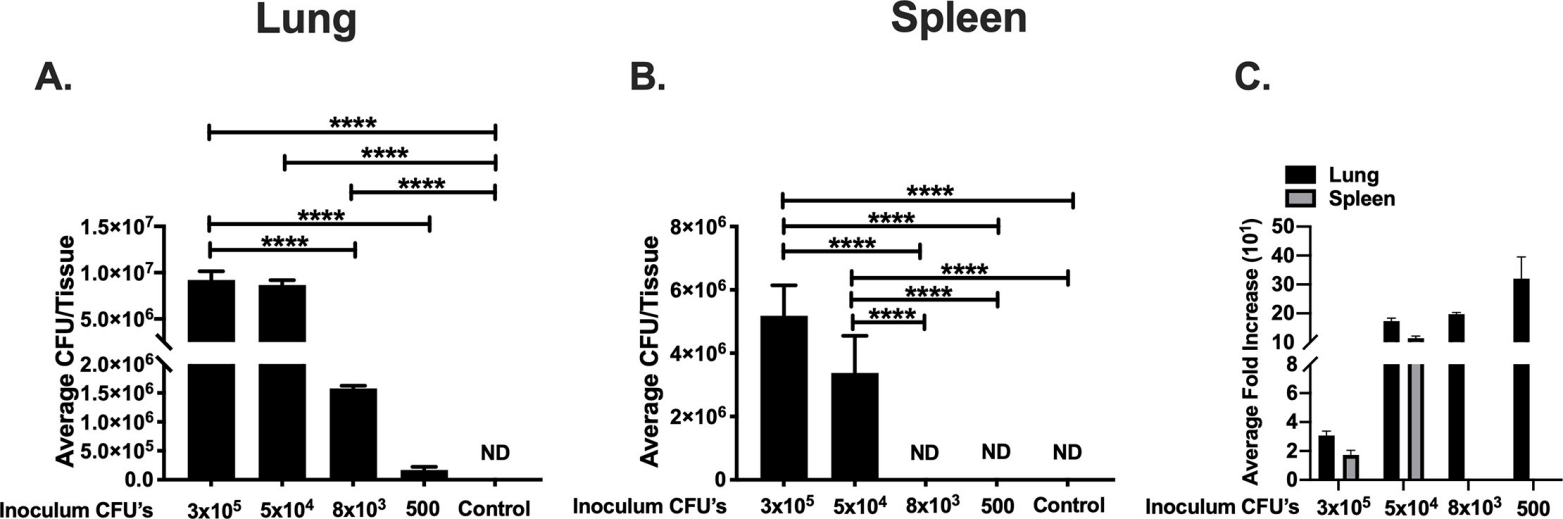
Bacterial load in lung and spleen of binge-drinking mice intranasally infected with decreasing doses of *B*. *thailandensis*. Colony forming units (CFUs) per lung **(A)**. Colony forming units (CFUs) per spleen **(B)**. C57BL/6 mice were administered alcohol (4.4 g/kg) or PBS intraperitoneally (i.p.) and 0.5 h later mice were inoculated intranasally with *B*. *thailandensis* at doses of (3 x 10^5^, 5 x 10^4^, 8 x 10^3^, or 500). Tissues were collected 24 h post infection and bacterial tissue burden was determined (CFU/Tissue). Each bar represents the mean of each group inoculated with a respective dose with SD, N = 6 per group. Average fold increase comparison of lung and spleen tissue **(C)**. Fold increase is based on initial bacterial dose and mean final bacterial burden with SD. ND = Not Detected; no bacteria was cultured on any LB media plate. (Control) indicates infected mice (3 x 10^5^ CFU) not administered alcohol. Horizontal lines and asterisks (*) represent statistical comparison of (10^5^ dose) and subsequent lower doses by Student’s *t*-test with Welch’s correction. ****, p ≤ 0.0001.

To assess *B*. *thailandensis* dose dependent effect on bacterial dissemination during a single binge alcohol episode, C57BL/6 mice were administered a single binge alcohol dose i.p. 0.5 h prior to an intranasal infection of decreasing bacterial CFUs. Lung and spleen tissues were harvested to measure bacterial burden in localized and distal tissue receptively. No bacteria were detected in lung or spleen tissue of mice infected and not administered alcohol. Mice administered alcohol and infected with the highest bacterial dose (3 X 10^5^) were burdened with ~ 1 x 10^7^ CFUs in lung tissue, with no significant change in lung bacterial burden when mice were inoculated with a decreased dose of (5 x 10^4^) CFUs and alcohol. Mice inoculated with (8 x 10^3^) CFU’s and alcohol were burdened with ~ 1.5 x 10^6^, a ~ 7-fold decrease in lung CFUs compared to the 3 x 10^5^ dose. Interestingly, mice infected with the lowest dose (500) CFUs and alcohol still harbored ~ 1.6 x 10^5^ CFUs in lung tissue 24 h PI (Fig 1A).

We then assessed *B*. *thailandensis* dissemination to the spleen with binge alcohol exposure. Mice that were administered a single binge alcohol dose 0.5 h prior to the highest infectious dose (3 x 10^5^) or lower (5 x 10^4^) CFUs, exhibited ~ 6 x 10^6^ CFUs and ~ 4 x 10^6^ CFUs in spleen tissue respectively. No statistical difference was measured in spleen tissue burden from the 3 x 10^5^ or 5 x 10^4^ CFU dose. Interestingly, bacteria were not detected in spleen tissue of mice inoculated with (8 x 10^3^ or 500) CFUs and administered alcohol. *B*. *thailandensis* was detected in whole blood of mice administered alcohol and 3 x 10^5^ or 5 x 10^4^ CFUs 24 h PI (S4 Table).

To further investigate bacterial persistence in the lungs even after a single low inoculation dose of 500 CFUs, bacterial fold changes utilizing initial inoculation dose and final bacterial burden was determined in mice that received the alcohol treatment. Intriguingly, mice inoculated with low (5 x 10^4^ or 8 x 10^3^) CFUs exhibited a significantly higher pulmonary bacterial fold increase compared to mice infected with the higher (3 x 10^5^) inoculation dose. Mice infected with the lowest inoculation dose of 500 CFU’s experienced the greatest pulmonary bacterial fold increase. A similar pattern was measured in spleen tissue, when mice were inoculated with (5 x 10^4^) compared to the higher initial dose (3 x 10^5^) CFU’s (Fig 1C). Moreover, a 12% decrease in body weight was measured in mice infected with a low dose of 500 CFUs compared to a 10.7% reduction in weight when mice were infected with 8 x 10^3^ CFUs. Mice infected with a single dose of 3 x10^5^ or 5 x 10^4^ experienced a reduction in body weight of 8.8 and 8.7% respectively (S1 Table). Thus, these data suggest that a single administration of alcohol decreases the infectious dose for dissemination and increases bacterial persistence in lung tissue after a very low inoculum dose.

### *B*. *thailandensis* persists in lung tissue after a very low dose of alcohol is administered 0.5 h prior to infection

**Fig 2:**
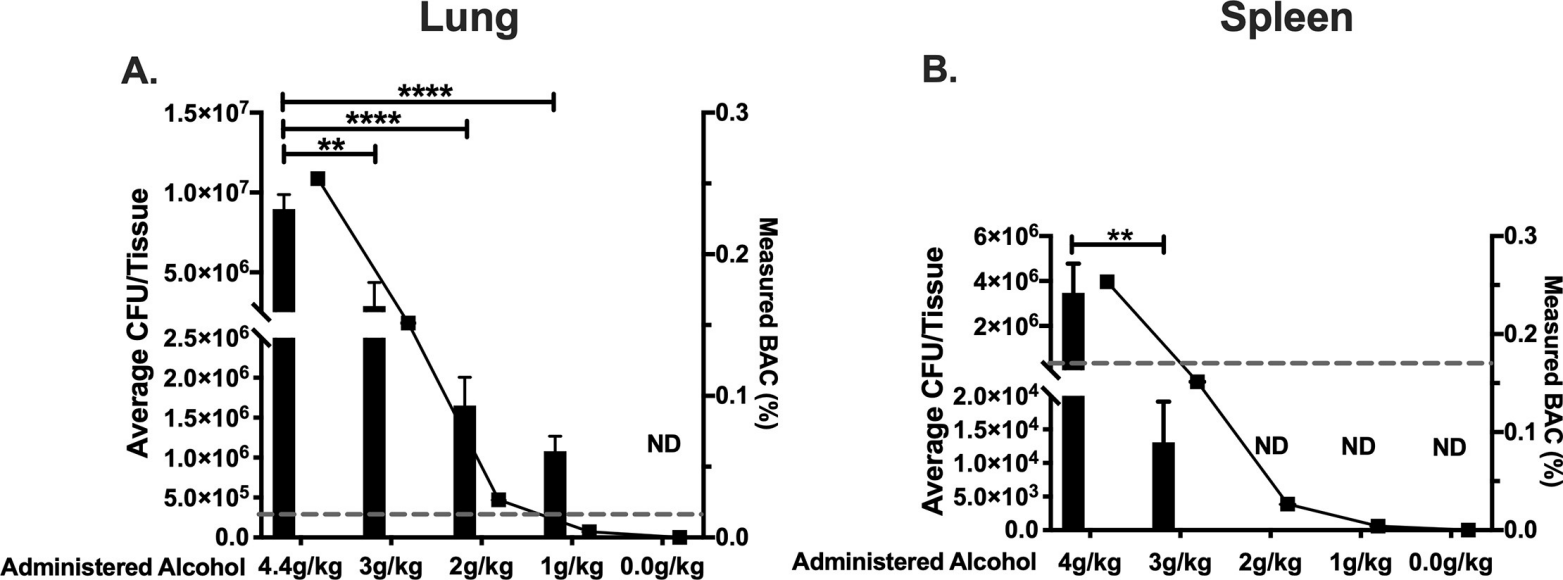
Bacterial load in lung and spleen of mice intranasally infected with *B*. *thailandensis* and administered different alcohol doses. Colony forming units (CFUs) per lung **(A)**. Colony forming units (CFUs) per spleen **(B)**. C57BL/6 mice were administered alcohol at doses of (4.4, 3, 2, 1 g/kg) or PBS intraperitoneally (i.p.) and 0.5 h later mice were inoculated intranasally with *B*. *thailandensis*. Dashed line represents the inoculating bacterial dose of (3 x 10^5^) CFUs. Tissues were collected 24 h post infection and bacterial tissue burden was determined (CFU/Tissue). Each bar represents the mean of each group administered a respective alcohol dose with SD, N = 6 per group. Measured BAC (%) indicated with () and line represents average BAC measured from blood collected 0.5 h prior to infection from each group (0.254, 0.152, 0.0265, 0.00397%) or PBS respectively. ND = Not Detected; no bacteria was cultured on any LB media plate. Horizontal lines and asterisks (*) represent statistical comparison of (4.4 g/kg dose) and subsequent lower alcohol doses by Unpaired Student’s *t*-test. **, p ≤ 0.01, ****, p ≤ 0.0001.

The quantity and concentration of alcohol consumed are critical factors in determining physiological impact of tissues. To assess the effects of alcohol concentration on *B*. *thailandensis* tissue dissemination during a single binge alcohol episode, C57BL/6 mice were administered a single binge alcohol dose at decreasing alcohol concentrations i.p., 0.5 h prior to a single intranasal infection of 3 x 10^5^ CFUs. Lung and spleen tissues were harvested to measure bacterial burden. No bacteria were detected in lung or spleen tissue of mice infected and not administered alcohol. Mice infected and administered 4.4 g/kg of alcohol (0.254, BAC) were burdened with ~ 1 x 10^7^ CFUs in lung tissue. Infected mice administered 3 g/kg of alcohol (0.152, BAC) were burdened with ~ 5 x 10^6^, a ~ 2-fold decrease in lung CFUs compared to mice administered a higher concentration of alcohol (4.4 g/kg). Remarkably, mice infected and administered 2 g/kg (0.0264, BAC) or 1 g/kg (0.00397, BAC) still harbored ~ 1.5 x 10^6^ and 1 x 10^6^ CFUs respectively in lung tissue 24 h PI (Fig 2A).

*B*. *thailandensis* dissemination to the spleen was assessed when mice were exposed to different alcohol concentrations. Mice that were administered a single infectious dose 0.5 h after the highest binge alcohol dose (4.4 g/kg) or lower (3 g/kg), exhibited ~ 4 x 10^6^ CFUs and ~ 1.5 x 10^4^ CFUs in spleen tissue respectively. Interestingly, bacteria were not detected in spleen tissue of mice administered (2 g/kg or 1 g/kg) alcohol. *B*. *thailandensis* was detected in whole blood of infected mice whose BAC reached 0.254 or 0.152 (S4 Table).

Furthermore, an average of 9.2% decrease in body weight was measured within infected mice administered 4.4 g/kg alcohol compared to an 8.2% reduction when mice were administered 3 g/kg. Mice infected with a single dose of 3 x10^5^ CFUs and administered 2 g/kg or 1 g/kg exhibited a reduction in body weight of 7.4 and 5.2% respectively (S2 Table). These data indicate that bacteria persist in lung tissue after a single very low alcohol dose is administered 0.5 h prior to infection.

### *B*. *thailandensis* persists in lung tissue after alcohol dose is administered 24 h prior to infection

**Fig 3:**
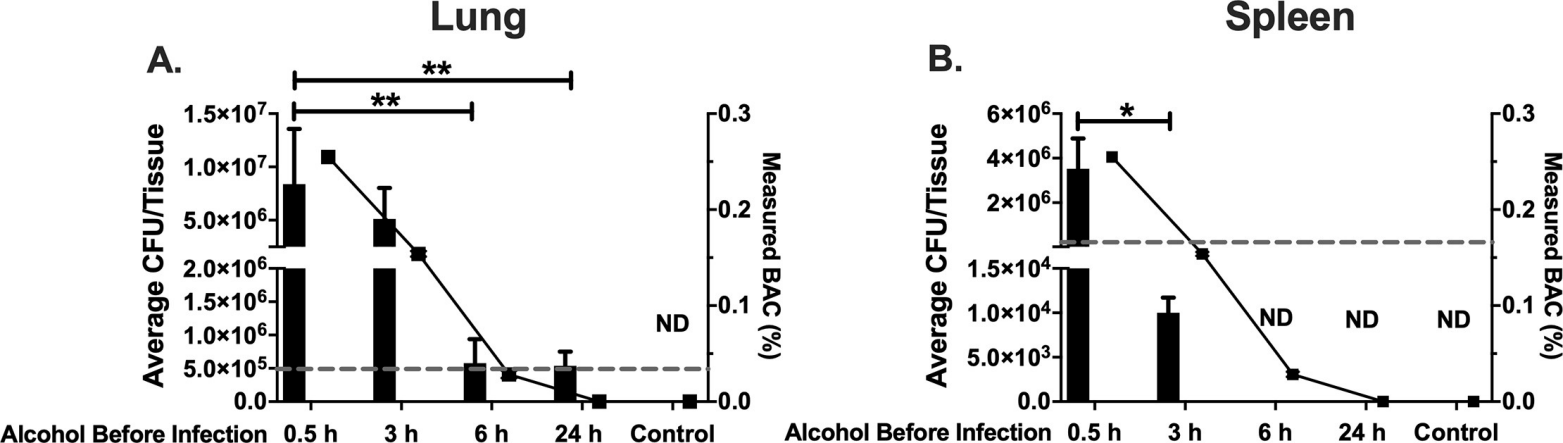
Bacterial load in lung and spleen of mice intranasally infected with *B*. *thailandensis* and temporal effects of alcohol during infection. Colony forming units (CFUs) per lung **(A).** Colony forming units (CFUs) per spleen **(B).** C57BL/6 mice were administered alcohol (4.4 g/kg) or PBS intraperitoneally (i.p.) and (0.5, 3, 6, or 24 h) later mice were inoculated intranasally with *B*. *thailandensis*. Dashed line represents the inoculating bacterial dose of (5 x 10^5^) CFUs. Tissues were collected 24 h post infection and bacterial tissue burden was determined (CFU/Tissue). Each bar represents the mean of each group administered alcohol at a respective time prior to infection with SD, N = 6 per group. Measured BAC (%) indicated with () and line represents average BAC measured from blood collected 0.5, 3, 6, or 24 h prior to infection from each group (0.254, 0.156, 0.0312, 0.0%) or PBS respectively. ND = Not Detected; no bacteria was cultured on any LB media plate. (Control) indicates infected and BAC 0.0%. Horizontal lines and asterisks (*) represent statistical comparison of (0.5 h alcohol prior to infection) and subsequent alcohol exposure prior to infection by Unpaired Student’s *t*-test, *, p ≤ 0.05, **, p ≤ 0.01.

The temporal effects of binge alcohol intoxication and bacterial–host contact time are of great interest from a clinical and public health perspective. To assess the temporal effects of a single binge alcohol episode on *B*. *thailandensis* tissue dissemination, C57BL/6 mice were administered a single binge alcohol dose (4.4 g/kg) i.p., 0.5, 3, 6, or 24 h prior to a single intranasal infection of 5 x 10^5^ CFUs. Lung and spleen tissues were harvested to measure bacterial burden. No bacteria were detected in lung or spleen tissue of mice infected and not administered alcohol. Mice administered 4.4 g/kg of alcohol (0.254, BAC) 0.5 h prior to infection were burdened with ~ 1 x 10^7^ CFUs in lung tissue. Mice administered alcohol 3 h prior to infection were burdened with ~ 5 x 10^6^, a ~ 2-fold decrease in lung CFUs compared to mice administered alcohol closer to the time of infection (0.5 h). Remarkably, mice administered alcohol 6 or 24 h prior to infection still harbored ~ 8 x 10^5^ and 5 x 10^5^ CFUs respectively in lung tissue (Fig 3A).

To assess *B*. *thailandensis* dissemination to the spleen, mice were exposed to alcohol at different time intervals prior to infection. Mice that were administered a single infectious dose 0.5 or 3 h after the binge alcohol dose (4.4 g/kg) exhibited ~ 5 x 10^6^ and ~ 2 x 10^4^ CFUs in spleen tissue respectively. Bacteria were not detected in spleen tissue of mice administered alcohol 6 or 24 h prior to infection. *B*. *thailandensis* was detected in whole blood of infected mice who received alcohol 0.5 or 3 h prior to infection (S4 Table).

Moreover, an average of 9.3% decrease in body weight was measured within mice administered 4.4 g/kg alcohol 0.5 h prior to infection. No statistical difference in weight loss was measured in mice administered alcohol 0.5 or 3 h prior to infection. Interestingly, mice administered alcohol 6 or 24 h prior to infection exhibited a greater reduction in body weight of 10.8 and 9.7% respectively (S3 Table). Taken together, these data indicate that the temporal effects of a single binge alcohol episode persist in the lung microenvironment even when alcohol metabolism has occurred for an extended period of time.

### GM-CSF and TNF-α remain suppressed, while IL-12/p40 concentration improves in lung tissue as early as 24 h post alcohol administered in mice

**Fig 4:**
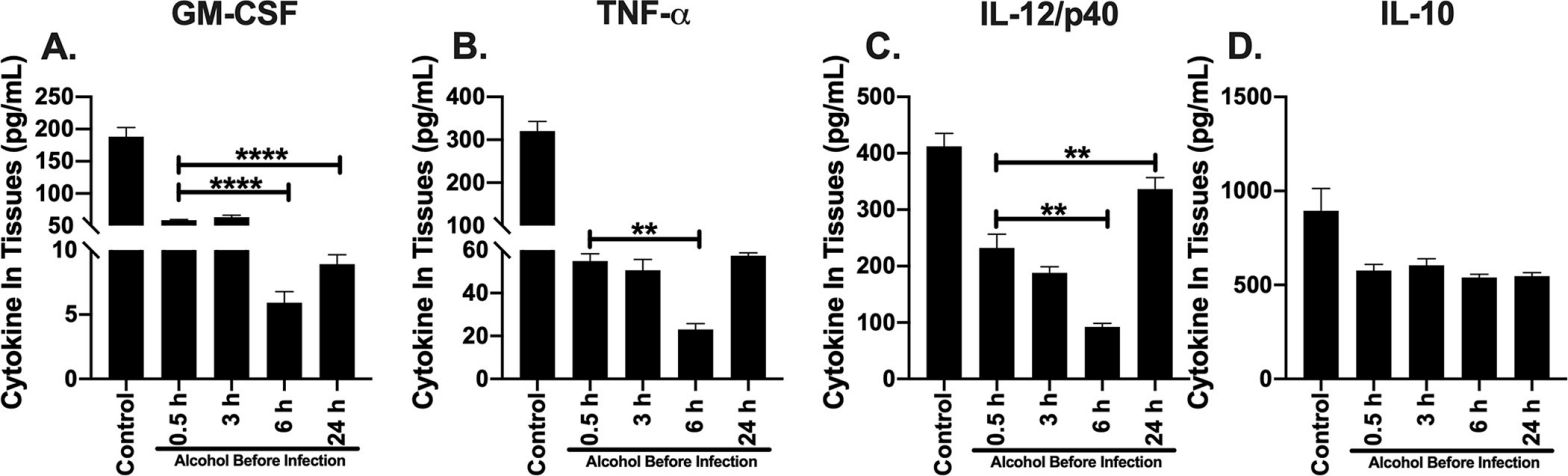
Cytokines in lung of mice intranasally infected with *B*. *thailandensis* and temporal effects of alcohol during infection. GM-CSF **(A)**. TNF-α **(B)**. IL-12/p40 **(C)**. IL-10 per lung **(D)**. C57BL/6 mice were administered alcohol (4.4 g/kg) or PBS intraperitoneally (i.p.) and (0.5, 3, 6, or 24 h) later mice were inoculated intranasally with *B*. *thailandensis* (5 x 10^5^) CFUs. Tissues were collected 24 h post infection and cytokine concentration determined by ELISA with corresponding protein standard. Each bar represents the mean of each group administered alcohol at a respective time prior to infection with SD, N = 6 per group. (Control) indicates infected mice not administered alcohol. Horizontal lines and asterisks (*) represent statistical comparison of (0.5 h alcohol prior to infection) and subsequent alcohol exposure prior to infection by one-way ANOVA **, p ≤ 0.01, ****, p ≤ .0001.

To further investigate the persistence of bacteria in lung tissue after alcohol was administered 6 or 24 h prior to infection, we measured the cytokine profile of the lung microenvironment of infected mice administered alcohol temporally. We used an ELISA as an indicator of GM-CSF, TNF-α, IL-12/p40 and IL-10 concentrations per lung tissue. All cytokines were decreased in mice administered a single dose of alcohol (4.4g/kg or PBS) compared to mice not administered alcohol (Fig 4). Mice administered binge alcohol 0.5 or 3 h prior to infection did not exhibit a significant difference in GM-CSF, TNF-α, IL-12/p40 or IL-10 concentrations in lung tissue homogenates (Fig 4). Conversely, GM-CSF concentrations were suppressed 2-fold in mice administered alcohol 6 or 24 h prior to infection compared to mice administered alcohol 0.5 h prior to infection (Fig 4A). Similarly, TNF-α was suppressed ~ 2-fold in mice administered alcohol 6 h prior to infection compared to mice administered alcohol 0.5 h prior to infection (Fig 4B). Remarkably, IL-12/p40 remained significantly suppressed in mice administered alcohol 6 h prior to infection compared to alcohol administration 0.5 h prior to infection, while mice administered alcohol 24 h prior to infection exhibited a ~ 2-fold increase in IL-12/p40 compared to mice given alcohol 0.5 h prior to infection. No statistical difference was measured in IL-12/p40 between mice administered alcohol 24 h prior to infection and mice not administered alcohol (control) (Fig 4C). Although not statistically significant, IL-10 was lower in mice administered alcohol 6 or 24 h prior to infection compared to alcohol administration 0.5 or 3 h prior to infection. IL-10 was neither significantly different among mice who received alcohol or compared to non-alcohol administered mice (Fig 4D). In combination, these results suggest that the temporal effects of alcohol may affect different distinct cell signaling pathways and elevated IL-12/p40 could lead to protective mechanisms that further reduce bacteria in the lung.

### IL-12/p40 expression improves in spleen tissue as early as 6 h post alcohol administered in mice

**Fig 5:**
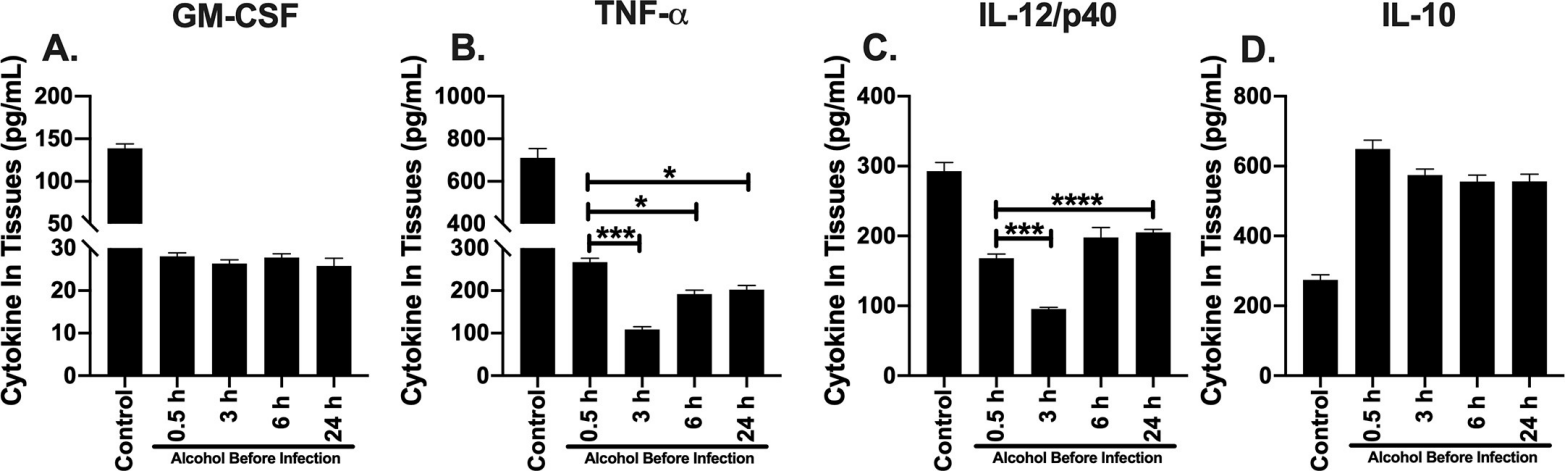
Cytokines in spleen of mice intranasally infected with *B*. *thailandensis* and temporal effects of alcohol during infection. GM-CSF **(A)**. TNF-α **(B)**. IL-12/p40 **(C)**. IL-10 **(D)**. C57BL/6 mice were administered alcohol (4.4 g/kg) or PBS intraperitoneally (i.p.) and (0.5, 3, 6, or 24 h) later mice were inoculated intranasally with *B*. *thailandensis* (5 x 10^5^) CFUs. Tissues were collected 24 h post infection and cytokine concentration determined by ELISA with corresponding protein standard. Each bar represents the mean of each group administered alcohol at a respective time prior to infection with SD, N = 6 per group. (Control) indicates infected mice not administered alcohol. Horizontal lines and asterisks (*) represent statistical comparison of (0.5 h alcohol prior to infection) and subsequent alcohol exposure prior to infection by one-way ANOVA, *, p ≤ 0.05, ***, p ≤ 0.001, ****, p ≤ .0001.

To further investigate bacteria clearance in spleen tissue after alcohol was administered 6 or 24 h prior to infection, we measured the cytokine profile of the spleen microenvironment of infected mice administered alcohol temporally. The intent of this assay was to characterize the effects of the temporal changes of a single bout of binge alcohol intoxication on important regulatory cytokines that may further inform about the differences observed between innate immune mediated bacterial clearance of lung and spleen tissue. We used an ELISA as an indicator of GM-CSF, TNF-α, IL-12/p40 and IL-10 concentrations per spleen tissue. GM-CSF, TNF-α, and IL-12/p40 cytokines were decreased and IL-10 increased in mice administered a single dose of alcohol (4.4g/kg or PBS) compared to mice not administered alcohol (Fig 5). Mice administered binge alcohol 0.5, 3, 6, or 24 h prior to infection did not exhibit a significant difference in GM-CSF, concentrations in spleen tissue homogenates (Fig 5A). Conversely, TNF-α and IL-12/p40 concentrations were suppressed ~ 2-fold in mice administered alcohol 3 h prior to infection compared to mice administered alcohol 0.5 h prior to infection (Fig 5B). Similarly, TNF-α was significantly suppressed in mice administered alcohol 6 or 24 h prior to infection compared to mice administered alcohol 0.5 h prior to infection (Fig 5B). Remarkably, IL-12/p40 was elevated in mice administered alcohol as early as 6 h prior to infection compared to alcohol administration 0.5 h prior to infection, while mice administered alcohol 24 h prior to infection exhibited a ~ 2-fold increase in IL-12/p40 compared to mice given alcohol 0.5 h prior to infection (Fig 5C). Although IL-10 was elevated in mice administered alcohol compared to control mice not administered alcohol, no statistical difference in IL-10 was measured among the groups of mice administered alcohol at any time prior to infection. (Fig 5D). These results indicate that IL-12/p40 expression could be tissue specific, with IL-12/p40 in spleen tissue possibly reducing the temporal effects of alcohol earlier when compared to lung tissue.

### Binge alcohol increases intracellular invasion of non-phagocytic human lung epithelial cells

**Fig 6:**
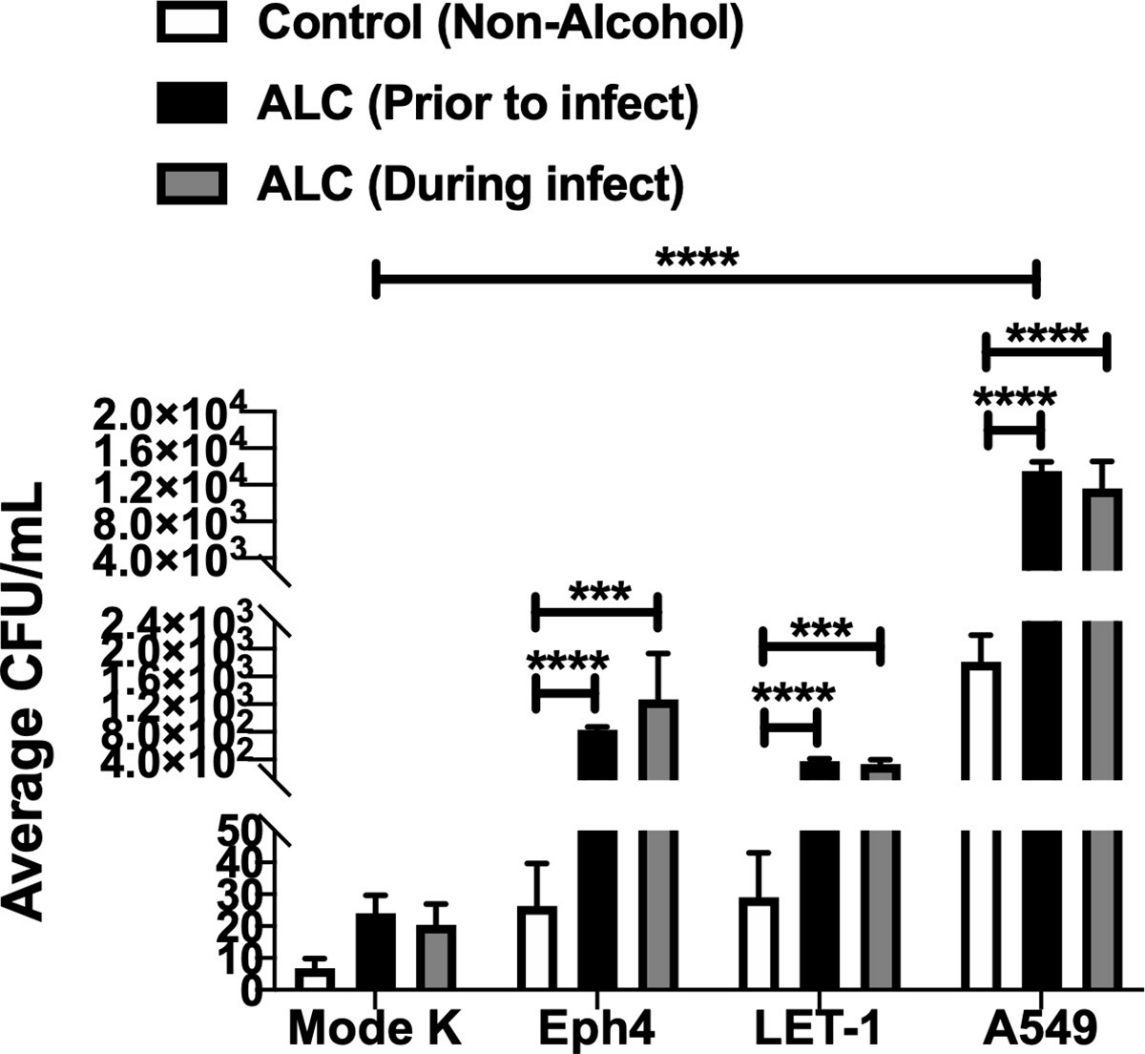
*B*. *thailandensis* invasion and survival in non-phagocytic lung and intestinal cells with and without alcohol treatment. Murine (Eph4, LET-1), intestinal (Mode K), or human (A549) lung epithelial cells were grown to confluency in cell culture media and co-cultured with *B*. *thailandensis* (MOI 1:10) with 0.0% or 0.2% v/v alcohol. ALC (Prior to infect) indicates cells treated with alcohol for 3 h prior to infection. ALC (During infect) indicates cells treated with alcohol at the time of infection. All cells were co-cultured for 3 h post alcohol treatment. Extracellular bacteria were removed by washes X4 and antibiotic treatment for 2 h. Cells were lysed, and viable bacteria recovered. Asterisks (*) represent statistical comparisons between ALC (Prior to infect) or (During infect) treatment and (Control, Non-Alcohol) determined by two-way ANOVA and Dunnett’s multiple comparisons test. Bars represent average CFU with SD. ***, p ≤ 0.001, ****, p ≤ 0.0001. Group comparison to Mode K cells determined by two-way ANOVA and Tukey’s multiple comparison test, **** p ≤ 0.0001, N = 3.

To further study the susceptibility of non-phagocytic lung epithelial cells to bacterial invasion after a binge alcohol dose, we developed an *in vitro* model to test 2 murine (Eph4 and LET1) and 1 human lung epithelial cell types for susceptibility to invasion. A murine intestinal (Mode K) non-lung epithelial cell line was used as a comparative control cell type to test the intracellular invasion potential for *B*. *thailandensis* in a distantly related epithelial cell compared to the lung. Monolayers were formed and co-cultured with or without alcohol. The results in Fig 6 show the average number of CFUs, demonstrating viable intracellular *B*. *thailandensis* isolated 3 h after challenge. All cell types incubated in alcohol 3 h prior to infection exhibited greater intracellular invasion compared to non-alcohol treated cells (Fig 6). Although not statistically different when comparing alcohol and non-alcohol treated intestinal cells, Mode K cells incubated in alcohol prior to infection were invaded with ~ 3-fold less bacteria compared to bacterial invasion of tested lung cell types exposed to alcohol. LET1 cells are a type 1 lung epithelial cell that constitute ~ 90% of the alveolar surface during gas exchange between alveoli and blood [21]. LET1 cells incubated in alcohol prior to infection were invaded with ~3-fold more bacteria than LET1 cells not incubated in alcohol. Similarly, Eph4 are cells found in the blood-air barrier and are critical to the pathogen response in the lung [22]. Eph4 cells incubated in alcohol prior to infection were invaded by bacteria ~ 3-fold more than cells not incubated in alcohol. Interestingly, A549 type 2 human epithelial cells that constitute ~ 60% of cells in the lung and can also be found in the blood-air barrier, were invaded with the greatest number (~ 1.5 x 10^4^) of bacteria when cells were incubated in alcohol prior to infection. Comparable with the mode of alcohol administration described in our mouse model, epithelial cells were also incubated in alcohol at the time of infection with similar trends observed when alcohol was administered prior to epithelial infection. When lung cells was compared as a group to intestinal Mode K cells, only the human lung epithelial cells were significantly different (Fig 6). These findings suggest a single binge alcohol episode increases bacterial survival and dissemination to a greater extent in lung tissue, at least in part, through an increase in intracellular invasion of non-phagocytic epithelial cells.

## Discussion

Binge alcohol consumption has immunomodulatory effects, which result in changes in morbidity and mortality rates associated with acute and chronic diseases, including infection [23, 24]. Our previous studies have indicated that a single binge alcohol episode of C57BL/6 mice before intranasal infection increased infectivity of less pathogenic *B*. *thailandensis*, in part by decreasing phagocytic mechanisms and altering epithelial cells, therefore increasing paracellular diffusion and intracellular invasion [11]. Melioidosis is linked to binge alcohol use, but the extent to which bacterial and alcohol dosage play a significant role in disease progression have not been examined. In addition, the temporal effects of a single binge alcohol dose prior to a *B*. *pseudomallei* near-neighbor infection has not been examined *in vivo*. Our hypothesis was that by decreasing the intranasal inoculum dose or the blood alcohol concentration, the host innate immune response would clear the bacteria from tissues by 24 h post infection. In addition, we hypothesized that a lone binge alcohol dose administered more than 6 h prior to infection would result in less bacterial colonization in comparison to earlier alcohol dosing and clear the infection in lung and spleen tissue, given no blood alcohol is detectable beyond this point in our mouse model.

In the present study we used less-pathogenic *B*. *thailandensis* E264, as a model to study the interplay between binge alcohol exposure and bacterial dose on tissue colonization in mice. *B*. *thailandensis* tissue colonization at the highest dose (5 x 10^5^ CFU) did not occur in mice that only received the vehicle solution (non-alcohol). Comparisons of alcohol induced tissue colonization and vehicle controls are indicated in Fig 1. Rather, comparisons among alcohol groups indicate an alcohol related effect on CFU’s required to colonize spleen and lung tissue. Moreover, bacterial colonization and persistence of lung and spleen tissue after a reduction in inoculum dose where BAC of 0.254% was obtained indicates an alcohol dependent effect that significantly reduces the number of *B*. *thailandensis* required to colonize vital organs in mice (Fig 1A and 1B). Interestingly, when the inoculum dose was decreased to 500 CFUs, total viable bacteria recovery was decreased while bacterial replication and survival was significantly increased when compared to all other initial inoculum doses and viable bacteria were collected from lung tissue 24 h post infection (Fig 1C). Furthermore, bacteria remained in whole blood when inoculum CFUs were reduced from 3 x 10^5^ to 5 x 10^4^, compared to no detectable bacteria in spleen tissue and whole blood when mice were inoculated with ≤ 8 x 10^3^ CFUs and BAC reached 0.254% prior to infection.

Inflammatory infiltrates in C57BL/6 mouse spleen tissue may explain, in part, the clearance of *B*. *thailandensis* from the spleen compared to persistent colonization in lung tissue. Similarly, other groups using C57BL/6 models have reported that infiltration of several different splenic macrophage phenotypes and T-cells that may serve to contain bacteria in the spleen, promoting an adequate localized immune response that reduces bacterial colonization in the spleen compared to bacterial persistence in lung tissue [25]. Likewise, *B*. *thailandensis* may be less able to efficiently colonize the spleen compared to more virulent *B*. *pseudomallei*, particularly after intranasal challenge [26]. It is plausible that low competition and higher available nutrients contributed to the increase in replication and overall persistence in alcohol infused lung tissue from a low initial 500 CFU infection [27]. The ability of *B*. *thailandensis* to colonize the lungs in mice not administered alcohol is dose dependent and substantially decreased when compared to *B*. *pseudomallei* [28]. C57BL/6 mice are more resistant to *Burkholderia* spp. intranasal infections then BALB/c mice, yet ≥ 10^6^ CFUs of *B*. *thailandensis* is generally required compared to ≤ 10^3^ CFUs of *B*. *pseudomallei* for pulmonary colonization [29].

Moreover, factors such as route of administration, inoculum dose, and virulence of the challenge organism are likely to influence host pathogen recognition and ultimately tissue colonization. Related findings indicate body weight (BW) can be used independently as a marker for disease severity or a moribund state in a variety of infectious disease models [30]. In our present study, a maximum of 12.3% reduction in BW was obtained from mice administered a single binge alcohol dose and infected with 500 CFUs, when compared to weight prior to infection (S1 Table). Mice exposed to a singular alcohol dose and infected with *B*. *thailandensis* at 5 x 10^4^ CFUs exhibited greater bacterial colonization of the lung and spleen at 24 h post infection and retained significant bacterial loads in the lung when infected with 8 x 10^3^ CFUs, compared to mice not administered alcohol. Considering the tolerance of C57BL/6 mice to *Burkholderia* species infections and the low virulence of *B*. *thailandensis*, these findings reveal that a single binge alcohol intoxication episode can increase tissue colonization while reducing the infectious dose required to colonize lung and spleen tissue with a less pathogenic *B*. *pseudomallei* near-neighbor. A more complete understanding of the effects of binge alcohol on a genetically similar and less pathogenic *Burkholderia* strain would allow for the development of effective preventative strategies for highly virulent *B*. *pseudomallei*.

To better understand the modulatory effects of a single binge alcohol episode as it relates to BAC and tissue colonization, mice were intranasally infected with a single non-lethal dose of *B*. *thailandensis* and administered ≤ 4.4 g/kg alcohol 0.5 h prior to infection. We have observed previously that colonization of lung and spleen tissue persists after the infectious dose is reduced and BAC is 0.254% prior to infection. In the present study, we observed an alcohol dose dependent decrease in lung and spleen colonization in C57BL/6 mice when BACs reached 0.152, 0.0265, or 0.00397% prior to infection (Fig 2). Intriguingly, lung colonization persisted in mice when BACs reached 0.0265% or 0.00397% (Fig 2A). Still, bacteria remained in whole blood when BAC was reduced from 0.254 to 0.152%, compared to no detectable bacteria in spleen tissue and whole blood when mice BAC reached ≤ 0.0256% prior to infection (Fig 2B). Similar to the significant BW loss observed in mice infected with a minimum of 500 CFUs and BAC at 0.254%; in this study, increased BW loss was correlated with higher BACs (S2 Table).

Alcohol effects differ by tissue and organ. Absorbed alcohol is primarily metabolized in the liver, but other tissues such as gastrointestinal mucosa, brain, spleen, and the lungs, also metabolize alcohol [31]. Alcohol not metabolized by the liver can diffuse from bronchial circulation and vaporize into conducting airways [32]. Vaporized alcohol in the lungs may explain, in part, the inability of mice to clear *B*. *thailandensis* completely from the lungs after a very low BAC. Vaporized alcohol cycles back into the airway lining fluid, repeatedly affecting the airway epithelium and creating a recycling of high concentrations of alcohol that results in “multi-exposures” in lung tissue even after a single binge alcohol episode [33]. Although outside the scope of this current study, repeated vapor alcohol exposure in lung tissue could magnify the deleterious effects of very low alcohol exposure, exacerbating dysfunction in alveolar macrophages and signaling pathways from epithelial cells during infection [34]. We have shown previously that alveolar macrophages exhibit phagocytic dysfunction as early as 2 h after a low 0.08% v/v alcohol exposure *in vitro* [18]. In the current study, mice whose BAC reached 0.0265 and 0.00397% prior to infection, which correspond to ~ 3 and 20 times below the threshold for legal intoxication in the United Sates (i.e., 0.08%) respectively, retained bacteria in lung and not in spleen tissues. Interestingly, in a binge drinking rat model, splenic T cells and resident macrophages were not depleted presumably because the spleen is related to immunity which may provide a sustained response promoting self-protection against comorbid injuries [35]. Furthermore, it is likely that most resident cells in lung and spleen tissues express ethanol metabolism activity, but specific information on the metabolism and immunological effects of alcohol in various tissue types is largely lacking [36]. Together, these data support our previous data indicating very low alcohol exposure impairs gas exchange in the lungs, resulting in alveolar macrophage and epithelial barrier dysfunction, reflecting that variations in tissue or organ exposure to alcohol are organ specific during infection [37].

Alcohol and infectious doses are only part of the problem for people coming into contact with *B*. *pseudomallei* or related near-neighbors, because alcohol can also have harmful temporal effects on host immune function [38]. However, the temporal effects of alcohol on *B*. *thailandensis* tissue colonization are less clear than binge alcohol intoxication 0.5 h prior to infection. In the current study, mice were administered a single binge alcohol dose 0.5, 3, 6, or 24 h prior to intranasal infection with less pathogenic *B*. *thailandensis*. Mice administered alcohol 0.5 or 3 h prior to infection produced greater tissue burden and were more susceptible to bacterial colonization of the lung and spleen, compared to mice administered alcohol 6 or 24 h prior to infection (Fig 3). As alcohol was metabolized in lung tissue the corresponding BAC was decreased when mice were administered alcohol 0.5 h compared to 3 h prior to infection. The lower BAC observed in mice administered alcohol 3 h prior to infection was not sufficient to significantly minimize bacterial colonization and replication of lung tissue (Fig 3A). Although a similar pattern of colonization was observed in spleen tissue, the decrease in BAC corresponding to alcohol administered 3 h prior to infection facilitated a significant decrease in bacterial survival and replication (Fig 3B). Interestingly, bacteria remained detectable in lung and not spleen tissue in mice administered alcohol 6 or 24 h prior to infection. Although bacteria in lung tissue were not cultured in quantities indicative of replication from mice administered alcohol 6 or 24 h prior to infection, the inability to eliminate bacteria is supportive of detrimental effects on lung and not spleen tissue even when alcohol is no longer detected in the blood. Bacteria were cultured from whole blood in mice administered alcohol 0.5 or 3 h prior to infection, and not at 6 or 24 h prior to infection. Although not statistically significant, BW was decreased to a greater extent in mice administered alcohol 6 or 24 h prior to infection (S3 Table). It remains unclear how body weight loss in our study is directly or indirectly affected by alcohol intoxication and *B*. *thailandensis*.

The effects of alcohol on various tissues depend on its concentration in the blood over time. Alcohol intoxication at moderate levels (or only occasionally), results in metabolization of alcohol (ethanol) to acetaldehyde by alcohol dehydrogenase (ADH) and subsequently acetaldehyde is catabolized to acetate by aldehyde dehydrogenase (ALDH) in the cytosol and/or mitochondria of most mammalian cells [39]. Acetaldehyde and acetate produced from the metabolism of alcohol contribute to cell and tissue damage by inhibiting enzyme, microsomal protein, and microtubules secretion as well as various metabolic processes [40]. Moreover, in our study the deleterious effects of acetaldehyde accumulation in the lungs may be responsible, at least in part, for the increase in bacterial colonization and replication when mice were administered alcohol 0.5 and 3 h prior to infection [41, 42]. Intriguingly, Gram negative *Helicobacter pylori* has been shown to increase localized acetaldehyde through metabolism of low levels of alcohol by *H*. *pylori* ADH [43]. In the current study, mice inoculated with *B*. *thailandensis* closer in time to elevated BACs, developed increased bacterial replication in the lungs. We can speculate that a moderate *B*. *thailandensis* infection that is localized in the lungs may have an additive host-pathogen affect that augments the accumulation of acetaldehyde as alcohol is metabolized in the lungs [43].

Tissue specific differences in alcohol metabolism are due in large part to age, sex, and species [44]. Characterization of the *Adh* gene in C57BL/6 inbred mice has revealed that the ADH enzyme and associated isoforms exist at higher frequencies compared to BALB/cJ and other commonly used laboratory mice. Furthermore, alcohol metabolism differs in both tissue and substrate specificity in mice and humans alike [45]. These data suggest that differences in lung and spleen bacterial colonization observed in our alcohol temporal effects study further support differences described with specific tissue metabolic rates associated within the C57BL/6 mouse strain and sex [45]. Further studies emphasizing the role of acetaldehyde and host-alcohol interactions during infection and potential differences between sex will be important future endeavors.

To better understand tissue colonization and the temporal effects of binge alcohol on innate immunity, lung and spleen tissue cytokines were examined among the groups of mice that were administered alcohol in a time dependent manner. Furthermore, the duration of action of alcohol in mice is poorly understood. As such, we sought to study the tissue cytokine profile of four important immuno-regulatory proteins as alcohol is being metabolized over time. These data inform about the state of the immune response during a bacterial infection when alcohol is administered. The cytokine, GM-CSF has a dual role in augmenting the recruitment and activation of both neutrophils and macrophages that boosts the infection-fighting ability of host lung defenses [35, 37]. From our study, GM-CSF and modulating cytokines TNF-α and IL-10 were suppressed in mice administered alcohol 0.5 or 3 h prior to infection, compared to mice not administered alcohol (Fig 4). Interestingly, mice administered alcohol 6 or 24 h prior to infection exhibited a significant reduction in GM-CSF and TNF- α when alcohol was administered at 6 h, followed by an increase in both cytokines when alcohol was administered 24 h prior to infection (Fig 4A and 4B). IL-10 was not significantly altered regardless of when alcohol was administered prior to infection in lung (Fig 4D) or spleen (Fig 5D) tissue. Moreover, binge alcohol administered 6 h prior to infection similarly reduced IL-12/p40. Surprisingly, IL-12/p40 exhibited recovery effects when alcohol was administered 24 h prior to infection in lung and spleen tissue, compared to mice who received alcohol 0.5, 3, 6 h prior to infection or non-alcohol controls.

These tissue cytokine profiles indicate a significant reduction in activated neutrophils and macrophages in mice who were administered alcohol 0.5, or 3 h prior to infection with the greatest suppression of GM-CSF, TNF- α, and IL-12/p40 observed when alcohol was administered 6 h prior to infection [46]. Interestingly, elevated IL-12/p40 in lung tissue of mice given a singular binge alcohol dose 24 h prior to infection with *B*. *thailandensis* may provide insight into lung tissue recovery mechanisms. IL-12/p40 has been found to play an important role in *in vivo* protection against *Burkholderia* infections. IL-12 and associated subunits were found to be critical for IFN-γ production [47]. In the current study, lung GM-CSF, TNF- α cytokines remain suppressed, while 1L-12/p40 is significantly increased 24 h post infection and 48 h after alcohol was initially administered (24 h prior to infection). It is plausible that IL-12/p40 is critical for bacterial clearance after binge alcohol intoxication. Binge alcohol may directly or indirectly suppress critical cytokine production by altering innate immune cells and cell phenotypes unique to each tissue type [48]. Our data suggests the temporal effects of binge alcohol intoxication may also be tissue specific [49]. Unlike lung IL-12/p40 suppression in mice administered alcohol 6 h prior infection, the spleen of infected mice expressed elevated IL-12/p40, in addition to increased expression when mice were administered alcohol 24 h prior to infection (Fig 5C). A rapid IL-12/p40 response in spleen and not lung tissue suggests differences in tissue-alcohol interactions with *B*. *thailandensis* leading to bacterial clearance in the spleen and not the lung, even when alcohol is low or not detected in the blood. Furthermore, elevated IL-10 in the spleen may be indicative of increased immuno-regulation that may facilitate a more rapid recovery from the temporal effects of alcohol and improved bacterial clearance (Fig 5D). The tissue specific effects of binge alcohol and the modulating effects of cytokines remain to be elucidated and serve as an attractive area of future studies.

Remarkably, bacteria at low numbers were still observed in lung tissues of mice administered alcohol 6 or 24 h prior to infection with *B*. *thailandensis*. These observations lead us to develop an *in vitro* assay to test the effects of alcohol in a binge-like pattern on lung epithelial bacterial invasion. Intracellular invasion of host cells is a successful survival strategy of many Gram-negative bacteria [28]. Although *B*. *thailandensis* has been found intracellularly in non-phagocytic cells in murine lung and brain, a quantitative study of alcohol-induced invasion of non-phagocytic human epithelial cells has never been conducted [11]. Furthermore, to test a possible explanation for bacterial persistence in lung tissue even when alcohol is not detected in blood, intracellular invasion of murine and human epithelial cells was compared to intestinal epithelial cells. The results obtained in the present study demonstrated that binge alcohol significantly increased bacterial invasion of both human and murine lung epithelial cells compared to intestinal epithelial cells (Fig 6). Both type 1 murine (Eph4 and LET1) and type 2 human (A549) lung epithelial cell types were more susceptible to intracellular invasion when compared to intestinal epithelial cells (Mode K) regardless of alcohol or non-alcohol exposure. Greater lung epithelial and not intestinal cell susceptibility to bacterial invasion when no alcohol was administered is especially relevant, considering mice administered alcohol 6 or 24 h prior to infection had very low or no detectable alcohol in whole blood respectively (Fig 3). Furthermore, intestinal epithelial barrier integrity has a significant role in preventing bacterial translocation into the blood and other tissues [50]. Consistently, hazardous alcohol consumption in mice induces disruption of the colonic mucosal barrier that leads to leakage of bacterial toxins [51]. Although less well-characterized, related research confirms acute alcohol has a detrimental effect on gut-derived endotoxins leading to pulmonary injury via the gut-liver-lung axis [33]. In the present study, murine intestinal epithelial cells were more resistant to intracellular invasion of *B*. *thailandensis* (Fig 6). It is possible that binge alcohol directly influences greater bacterial colonization of the lung by facilitating invasion of lung epithelial cells compared to other tissues types. Intriguingly, moderate alcohol abuse may promote bacterial passage through intestinal epithelial tight junctions, rather than intestinal cell invasion [52]. However, further research is necessary to more fully delineate whether the impact of BAC, alcohol metabolism, or vapor alcohol on lung tissue colonization is caused by modifications to epithelial raft structures, allowing for greater attachment, invasion, and bacterial survival; or synergy between bacterial gut diffusion via tight junction dysfunction, aggravated by the gut-liver-lung axis [53, 54]. To this end, a greater emphasis will need to be on TEER and cell permeability to test tissue specific mechanistic effects of alcohol on bacterial invasion susceptibility. Likewise, utilizing different bacterial stains will provide greater insight to our findings.

The data from the present study provide an important framework for *B*. *pseudomallei* near-neighbor virulence when patients engage in hazardous alcohol consumption (Table 1). Our results showed after a single binge alcohol episode with a relatively high BAC, an intranasal infection with less-pathogenic *B*. *thailandensis* can increase its infectivity, even while decreasing the bacterial dose. When adjusting the alcohol dose, our findings indicate that a single binge alcohol episode increased *B*. *thailandensis* infectivity in the lungs to a greater extent compared to spleen, even after a very low BAC, suggesting there may be no safe alcohol dose. Furthermore, our data indicate that temporally a single binge alcohol episode effects lung bacterial colonization even after alcohol is no longer detected in the blood. Moreover, our findings provide novel insights into a possible mode of action for bacterial tissue colonization and dissemination during binge alcohol exposure. Our three novel mouse models support similar findings that binge alcohol creates tissue specific effects in a multi-hit process that requires system level and organ-organ interaction analysis in order to more fully understand the role of comorbidities such as binge alcohol intoxication co-occurring with *B*. *pseudomallei* or near-neighbor infections in humans [55]. The data from these studies support the public health responses being developed in melioidosis-endemic regions that emphasize the nature of alcohol consumption as a prime concern [3, 6]. Emphasis is being placed on the dangers of binge drinking, especially around disadvantaged communities with increased prevalence of AUDs and exposure to environmental *B*. *pseudomallei*.

## Supporting information

S1 Table Average body weight of binge drinking C57BL/6 mice intranasally infected with different bacterial doses. Mice were administered alcohol (4.4 g/kg) and 0.5 h later intranasally infected with various bacterial doses. Mice were weighed before infection and 24 h post infection. PBS (control) indicates mice that were not infected or administered alcohol. Alcohol (control) indicates mice that were administered alcohol and not infected. (*) indicates statistical comparison between pre-infection and post-infection (24 h) per group by Student’s unpaired *t*-test, *, p ≤ 0.05, **, p ≤ 0.01.
(PDF)

S2 Table Average body weight of C57BL/6 mice administered different binge-alcohol doses. Mice were administered alcohol at doses (4.4, 3, 2, 1 g/kg) and 0.5 h later intranasally infected with *B*. *thailandensis* (3 x 10^5^*)*. Mice were weighed before infection and 24 h post infection. PBS (control) indicates mice that were not infected or administered alcohol. Alcohol (control) indicates mice that were administered alcohol and not infected. (*) indicates statistical comparison between pre-infection and post-infection (24 h) per group by Student’s unpaired *t*-test, *, p ≤ 0.05, **, p ≤ 0.01.
(PDF)

S3 Table Average body weight of C57BL/6 mice administered binge-alcohol doses at different times prior to infection. Mice were administered alcohol (4.4 g/kg) and (0.5, 3, 6, or 24 h) later mice were intranasally infected with *B*. *thailandensis* (5 x 10^5^*)*. Mice were weighed before infection and 24 h post infection. PBS (control) indicates mice that were not infected or administered alcohol. Alcohol (control) indicates mice that were administered alcohol and not infected. (*) indicates statistical comparison between pre-infection and post-infection (24 h) per group by Student’s unpaired *t*-test, *, p ≤ 0.05, **, p ≤ 0.01, ***, p ≤ 0.001.
(PDF)

S4 Table Average *B*. *thailandensis* detected in C57BL/6 whole blood. Whole blood cultures were collected 24 h post infection via cardiac puncture and viable *B*. *thailandensis* E264 was grown on LB media plates to determine colony forming units (CFUs). *B*. *thailandensis* E264 colonies confirmed per plate and assay. Whole blood CFUs represent average number of colonies for each group within a specific experimental assay; 1. Bacterial dosage (inoculum CFUs), 2. Alcohol dosage (administered alcohol), or 3. Temporal effects (alcohol before infection).
(PDF)

## References

[1] Rush B. (1808). An inquiry into the effects of ardent spirits upon the human body and mind: With an account of the means of preventing, and of the remedies for curing them (4^th^ ed.). Philadelphia: Printed for Thomas Dobson; Archibald Bartram, printer.

[2] Moss M. (2005) Epidemiology of Sepsis: Race, Sex, and Chronic Alcohol Abuse. Clin Infect Dis. 41(1): S490–S497. 10.1086/432003 16237652

[3] Currie BJ. (2015) Melioidosis: Evolving Concepts in Epidemiology, Pathogenesis, and Treatment. Semin Respir Crit Care Med. 36: 111–125. 10.1055/s-0034-1398389 25643275

[4] Wiersinga WJ, Currie BJ, Peacock SJ. (2012) Melioidosis. N Engl J Med. 367: 1035–44. 10.1056/NEJMra1204699 22970946

[5] Glass M, Gee J, Steigerwalt A, Cavuoti D, Barton T, Hardy R, et al (2006) Pneumonia and septicemia caused by Burkholderia thailandensis in the United States. Journal of clinical microbiology. 44(12): 4601–4604. 10.1128/JCM.01585-06 17050819 PMC1698378

[6] Currie BJ, Jacups SP, Cheng AC, Fisher DA, Anstey NM, Huffam SE, et al (2004) Melioidosis epidemiology and risk factors from a prospective whole-population study in northern Australia. Trop Med Int Health. (11):1167–74. Available from: http://www.ncbi.nlm.nih.gov/pubmed/15548312 10.1111/j.1365-3156.2004.01328.x . 15548312

[7] Bhatty M, Pruett SB, Swiatlo E, Nanduri B. (2011) Alcohol abuse and Streptococcus pneumoniae infections: Consideration of virulence factors and impaired immune responses. Alcohol. Elsevier Inc. 45: 523–539. 10.1016/j.alcohol.2011.02.305 21827928 PMC3214648

[8] Bermudez LE, Young LS, Martinelli J, Petrofsky M. (1993) Exposure to ethanol up-regulates the expression of Mycobacterium avium complex proteins associated with bacterial virulence. J Infect Dis. 168: 961–968. 10.1093/infdis/168.4.961 8376842

[9] Camarena L, Bruno V, Euskirchen G, Poggio S, Snyder M. (2010) Molecular mechanisms of ethanol-induced pathogenesis revealed by RNA-sequencing. PLoS Pathog. 6(4): e1000834 10.1371/journal.ppat.1000834 20368969 PMC2848557

[10] Gordon SB, Irving GRB, Lawson R a, Lee ME, Read RC. (2000) Intracellular Trafficking and Killing of Streptococcus pneumoniae by Human Alveolar Macrophages Are Influenced by Opsonins. Infection and Immunity 68(4): 2286–2293. 10.1128/iai.68.4.2286-2293.2000 10722631 PMC97415

[11] Jimenez V, Moreno R, Settles E, Currie BJ, Keim P, Monroy FP. (2018) A mouse model of binge alcohol consumption and Burkholderia infection. PLoS One. 13(11): 1–19. 10.1371/journal.pone.0208061 30485380 PMC6261616

[12] Goral J, Karavitis J, Kovacs EJ. (2008) Exposure-dependent effects of ethanol on the innate immune system. Alcohol. 42(4): 237–247. 10.1016/j.alcohol.2008.02.003 18411007 PMC2453223

[13] National Institutes of Alcohol Abuse and Alcoholism. Drinking levels defined. Available at: https://www.niaaa.nih.gov/alcohol-health/overview-alcohol-consumption/moderate-binge-drinking. Accessed February, 2019.

[14] Pruett SB, Schwab C, Zheng Q, Fan R. (2004) Suppression of innate immunity by acute ethanol administration: a global perspective and a new mechanism beginning with inhibition of signaling through TLR3. J Immunol. 173: 2715–2724. 10.4049/jimmunol.173.4.2715 15294990

[15] Rendon JL, Janda BA, Bianco ME, Choudhry MA. (2012) Ethanol Exposure Suppresses Bone Marrow-Derived Dendritic Cell Inflammatory Responses Independent of TLR4 Expression. J Interf Cytokine Res. 32(9): 416–425. 10.1089/jir.2012.0005 22812678 PMC3438840

[16] Yeligar SM, Harris FL, Hart CM, Brown LAS. (2014) Glutathione attenuates ethanol-induced alveolar macrophage oxidative stress and dysfunction by down-regulating NADPH oxidases. Am J Physiol Lung Cell Mol Physiol. 306: L429–L441. 10.1152/ajplung.00159.2013 24441868 PMC3949056

[17] Yeligar SM, Chen MM, Kovacs EJ, Sisson JH, Burnham EL, Brown LA. (2016) Alcohol and lung injury and immunity. Alcohol. 55: 51–59. 10.1016/j.alcohol.2016.08.005 27788778 PMC5319482

[18] Jimenez V, Moreno R, Kaufman E, Hornstra H, Settles E, Currie BJ, et al (2017) Effects of binge alcohol exposure on Burkholderia thailandensis–alveolar macrophage interaction. Alcohol. Elsevier Ltd. 64: 55–63. 10.1016/j.alcohol.2017.04.004 28965656

[19] Bhatty M, Tan W, Basco M, Pruett S, Nanduri B. (2017) Binge alcohol consumption 18 h after induction of sepsis in a mouse model causes rapid overgrowth of bacteria, a cytokine storm, and decreased survival. Alcohol. 63:9–17. 10.1016/j.alcohol.2016.11.007 28847384 PMC8204640

[20] Eysseric H, Gonthier B, Soubeyran A, Bessard G, Saxod R, Barret L. (1997) There is no simple method to maintain a constant ethanol concentration in long-term cell culture: Keys to a solution applied to the survey of astrocytic ethanol absorption. Alcohol. 14: 111–115. 10.1016/s0741-8329(96)00112-7 9085710

[21] Diercks AH, Surman SL, Navarro G, Hurwitz JL, Rosenberger CM, Dash P, et al (2013) Characterization of innate responses to influenza virus infection in a novel lung type I epithelial cell model. J Gen Virol. 95: 350–362. 10.1099/vir.0.058438-0 24243730 PMC3917066

[22] Kwapiszewska G, Herold S, von Wulffen W, Cakarova L, Seeger W, Marsh LM, et al (2009) Surface expression of CD74 by type II alveolar epithelial cells: a potential mechanism for macrophage migration inhibitory factor-induced epithelial repair. Am J Physiol Cell Mol Physiol. 296: L442–L452. 10.1152/ajplung.00525.2007 19136583

[23] Nelson S, Kolls JK. (2002) Alcohol, host defense and society. Nat Rev Immunol. 2: 205–209. 10.1038/nri744 11913071

[24] Szabo G, Saha B. (2015) Alcohol’s Effect on Host Defense. Alcohol Res. 37 (2): 159–170. 26695755 10.35946/arcr.v37.2.01 PMC4590613

[25] D’Souza El-Guindy N, Kovacs EJ, De Witte P, Spies C, Littleton JM, De Villiers WJ et al (2010) Laboratory models available to study alcohol-induced organ damage and immune variations; choosing the appropriate model. Alcohol Clin Exp Res. 34(9): 997–1003. 10.1016/j.biotechadv.2011.08.021.Secreted 20586763 PMC2929290

[26] Morici LA, Heang J, Tate T, Didier PJ, Roy CJ. (2010) Differential susceptibility of inbred mouse strains to Burkholderia thailandensis aerosol infection. Microb Pathog. Elsevier Ltd. 48: 9–17. 10.1016/j.micpath.2009.10.004 19853031 PMC7006035

[27] Passalacqua KD, Charbonneau M-E, O’Riordan MXD. (2016) Bacterial Metabolism Shapes the Host–Pathogen Interface. Virulence Mechanisms of Bacterial Pathogens, Fifth Edition. 4(3): 1–31. 10.1128/microbiolspec.vmbf-0027-2015 27337445 PMC4922512

[28] Kespichayawattana W, Intachote P, Utaisincharoen P, Sirisinha S. (2004) Virulent Burkholderia pseudomallei is more efficient than avirulent Burkholderia thailandensis in invasion of and adherence to cultured human epithelial cells. Microb Pathog. 36: 287–292. 10.1016/j.micpath.2004.01.001 15043863

[29] Tan GYG, Liu Y, Sivalingam SP, Sim SH, Wang D, Paucod JC, et al (2008) Burkholderia pseudomallei aerosol infection results in differential inflammatory responses in BALB/c and C57BL/6 mice. J Med Microbiol. 57: 508–515. 10.1099/jmm.0.47596-0 18349373

[30] Trammell R, Toth LA. (2011) Markers for Predicting Death as an Outcome for Mice Used in Infectious Disease Research. Am Assoc Lab Anim Sci. 61(6): 492–498. 10.1111/j.2042-3306.1985.tb02052.x PMC3236690 22330575

[31] Romero F, Shah D, Hoek JB, Duong M, Lang CH, Stafstrom W, et al (2014) Chronic Alcohol Ingestion in Rats Alters Lung Metabolism, Promotes Lipid Accumulation, and Impairs Alveolar Macrophage Functions. Am J Respir Cell Mol Biol. 51(6): 840–849. 10.1165/rcmb.2014-0127OC 24940828 PMC4291549

[32] George SC, Hlastala MP, Souders JE, Babb AL. (1996) Gas Exchange in the Airways. J Aerosol Med. 9(1): 25–33. 10.1089/jam.1996.9.25 10172721

[33] Massey VL, Beier JI, Ritzenthaler JD, Roman J, Arteel GE. (2015) Potential role of the gut/liver/lung axis in alcohol-induced tissue pathology. Biomolecules. 5: 2477–2503. 10.3390/biom5042477 26437442 PMC4693244

[34] Simet S, Sisson J. Alcohol’s Effects on Lung Health and Immunity. (2015) Curr Rev. 37: 199–208. 10.35946/arcr.v37.2.05 PMC4590617 26695745

[35] Kim JS, Shukla SD. (2006) Acute in vivo effect of ethanol (binge drinking) on histone H3 modifications in rat tissues. Alcohol and Alcoholism. 41(2): 126–132. 10.1093/alcalc/agh248 16314425

[36] Kaphalia L, Calhoun WJ. (2013) Alcoholic lung injury: Metabolic, biochemical and immunological aspects. Toxicology Letters. 222(2): 1–21. 10.1016/j.toxlet.2013.07.016 23892124 PMC3806189

[37] Kershaw CD, Guidot DM. (2008) Putting Systems Biology Approaches Into Practice Alcoholic Lung Disease: Alcoholic lung disease. Alcohol Res Health. 31(1): 66–75. 23584753 PMC3860447

[38] Barr T, Helms C, Grant K, Messaoudi I. (2016) Opposing Effects of Alcohol on the Immune System Overview of the Immune System HHS Public Access. Prog Neuropsychopharmacol Biol Psychiatry. 65: 242–251. 10.1016/j.pnpbp.2015.09.001 26375241 PMC4911891

[39] Manzo-Avalos S, Saavedra-Molina A. (2010) Cellular and mitochondrial effects of alcohol consumption. International Journal of Environmental Research and Public Health. 7: 4281–4304 10.3390/ijerph7124281 21318009 PMC3037055

[40] Agarwal DP. (2001) Genetic polymorphisms of alcohol metabolizing enzymes. Pathol Biol. 49: 703–9. 10.1016/s0369-8114(01)00242-5 11762132

[41] Koivisto T, Salaspuro M. (1998) Acetaldehyde alters proliferation, differentiation and adhesion properties of human colon adenocarcinoma cell line Caco-2. Carcinogenesis. 19(11): 2031–2036. 10.1093/carcin/19.11.2031 9855020

[42] Mehta A, Guidot D. (2017) Alcohol and the lung. Alcohol Res Curr Rev. 38: 243–254. 10.35946/arcr.v38.2.07 PMC5513688 28988576

[43] Roine RP, Salmela KS, Salaspuro M. (1995) Alcohol metabolism in helicobacter pylori-infected stomach. Ann Med. 27(5): 583–588. 10.3109/07853899509002473 8541036

[44] Boule LA, Kovacs EJ. (2017) Alcohol, aging, and innate immunity. J Leukoc Biol. 102: 41–55. 10.1189/jlb.4RU1016-450R 28522597 PMC6608055

[45] Tussey L, Felder MR. (1989) Tissue-specific genetic variation in the level of mouse alcohol dehydrogenase is controlled transcriptionally in kidney and posttranscriptionally in liver. Proc Natl Acad Sci. 86: 5903–5907. 10.1073/pnas.86.15.5903 2474823 PMC297739

[46] Joshi PC, Applewhite L, Ritzenthaler JD, Roman J, Fernandez AL, Eaton DC, et al (2005) Chronic Ethanol Ingestion in Rats Decreases Expression and Downstream Signaling in the. 175: 6837–6845. 10.4049/jimmunol.175.10.6837 16272341

[47] Rowland CA, Lertmemongkolchai G, Bancroft, G. J.’Garra A, Bancroft A, Haque A, et al (2006) Critical Role of Type 1 Cytokines in Controlling Initial Infection with Burkholderia mallei. Infect Immun. 74: 5333–5430. 10.1128/IAI.02046-05 16926428 PMC1594859

[48] Bhatty M, Jan BL, Tan W, Pruett SB, Nanduri B. (2011) Role of acute ethanol exposure and TLR4 in early events of sepsis in a mouse model. Alcohol. 45: 795–803. 10.1016/j.alcohol.2011.07.003 21872420 PMC3293500

[49] Sibley D, Jerrells TR. (2000) Alcohol consumption by C57BL/6 mice is associated with depletion of lymphoid cells from the gut-associated lymphoid tissues and altered resistance to oral infections with Salmonella typhimurium. J Infect Dis. 182: 482–489. 10.1086/315728 10915079

[50] Wuest DM, Wing AM, Lee KH. (2013) Membrane configuration optimization for a murine in vitro blood-brain barrier model. J Neurosci Methods. 212: 211–221. 10.1016/j.jneumeth.2012.10.016 23131353

[51] Mir H, Meena AS, Chaudhry KK, Shukla PK, Gangwar R, Manda B, et al (2016) Occludin deficiency promotes ethanol-induced disruption of colonic epithelial junctions, gut barrier dysfunction and liver damage in mice. Biochim Biophys Acta—Gen Subj. 1860: 765–774. 10.1016/j.bbagen.2015.12.013 26721332 PMC4776745

[52] Wang Y, Zhang D, Wang B, Chang B, Wang B, Tong J. (2014) Effects of alcohol on intestinal epithelial barrier permeability and expression of tight junction-associated proteins. Mol Med Rep. 9: 2352–2356. 10.3892/mmr.2014.2126 24718485

[53] Patel S, Behara R, Swanson GR, Forsyth CB, Voigt RM, Keshavarzian A. (2015) Alcohol and the intestine. Biomolecules. 5: 2573–2588. 10.3390/biom5042573 26501334 PMC4693248

[54] Traphagen N, Tian Z, Allen-Gipson D. (2015) Chronic ethanol exposure: Pathogenesis of pulmonary disease and dysfunction. Biomolecules. 5: 2840–2853. 10.3390/biom5042840 26492278 PMC4693259

[55] Osna NA, Kharbanda KK. (2016) Multi-organ alcohol-related damage: Mechanisms and treatment. Biomolecules. 6(20): 1–5. 10.3390/biom6020020 27092531 PMC4919915

