# Supplementary material for: Persistence of Burkholderia thailandensis E264 in lung tissue after a single binge alcohol episode
Source: PLoS One. 2019 Dec 10;14(12):e0218147. doi: 10.1371/journal.pone.0218147 (PMC6903738; doi:10.1371/journal.pone.0218147)
Supplement: S1 Table — Mice were administered alcohol (4.4 g/kg) and 0.5 h later intranasally infected with various bacterial doses. Mice were weighed before infection and 24 h post infection. PBS (control) indicates mice that were not infected or administered alcohol. Alcohol (control) indicates mice that were administered alcohol and not infected. (*) indicates statistical comparison between pre-infection and post-infection (24 h) per group by Student’s unpaired t-test, *, p ≤ 0.05, **, p ≤ 0.01. (PDF) [file pone.0218147.s001.pdf]

S1 Table. Average body weight of binge drinking C57BL/6 mice intranasally infected with different bacterial doses. Mice were administered alcohol (4.4 g/kg) and 30 min later intranasally infected with various bacterial doses. Mice were weighed before infection and 24 h post infection. PBS (control) indicates mice that were not infected or administered alcohol. Alcohol (control) indicates mice that were administered alcohol and not infected. (*) indicates statistical comparison between pre-infection and post-infection (24 h) per group by Student's unpaired *t*-test, *, $p \leq 0.05$, **, $p \leq 0.01$.

	Body Weight (g)		
B. thailandensis (CFUs)	Pre-Infection	Post-Infection (24 h)	Decrease (%)
PBS (control)	19.8 ± 0.012 (n = 6)	19.8 ± 0.015 (n = 6)	0.0
Alcohol (control)	19.7 ± 0.121 (n = 6)	19.8 ± 0.132 (n = 6)	0.0
3×10^5	19.3 ± 0.327 (n = 6)	17.6 ± 0.230 (n = 6)	8.8 **
5×10^4	19.6 ± 0.528 (n = 6)	17.9 ± 0.478 (n = 6)	8.7 *
8×10^3	19.7 ± 0.448 (n = 6)	17.6 ± 0.412 (n = 6)	10.7 **
500	19.5 ± 0.351 (n = 6)	17.1 ± 0.444 (n = 6)	12.3 **

Values are means ± SEM; n, number of mice
