# Supplementary material for: Persistence of Burkholderia thailandensis E264 in lung tissue after a single binge alcohol episode
Source: PLoS One. 2019 Dec 10;14(12):e0218147. doi: 10.1371/journal.pone.0218147 (PMC6903738; doi:10.1371/journal.pone.0218147)
Supplement: S2 Table — Mice were administered alcohol at doses (4.4, 3, 2, 1 g/kg) and 0.5 h later intranasally infected with B. thailandensis (3 x 10^5^). Mice were weighed before infection and 24 h post infection. PBS (control) indicates mice that were not infected or administered alcohol. Alcohol (control) indicates mice that were administered alcohol and not infected. (*) indicates statistical comparison between pre-infection and post-infection (24 h) per group by Student’s unpaired t-test, *, p ≤ 0.05, **, p ≤ 0.01. (PDF) [file pone.0218147.s002.pdf]

S2 Table. Average body weight of C57BL/6 mice administered different binge-alcohol doses. Mice were administered alcohol at doses (4.4, 3, 2, 1 g/kg) and 30 min later intranasally infected with *B. thailandensis* (3 x 10^5). Mice were weighed before infection and 24 h post infection. PBS (control) indicates mice that were not infected or administered alcohol. Alcohol (control) indicates mice that were administered alcohol and not infected. (*) indicates statistical comparison between pre-infection and post-infection (24 h) per group by Student's unpaired *t*-test, *, $p \leq 0.05$, **, $p \leq 0.01$.

	Body Weight (g)		
BAC (%)	Pre-Infection	Post-Infection (24 h)	Decrease (%)
PBS (control)	19.3 ± 0.023 (n = 6)	19.3 ± 0.058 (n = 6)	0.0
Alcohol (control)	19.4 ± 0.098 (n = 6)	19.5 ± 0.131 (n = 6)	0.0
0.254	19.5 ± 0.312 (n = 6)	17.7 ± 0.241 (n = 6)	9.2 ***
0.152	19.3 ± 0.582 (n = 6)	17.7 ± 0.393 (n = 6)	8.2 *
0.0265	18.8 ± 0.412 (n = 6)	17.4 ± 0.300 (n = 6)	7.4 *
0.00397	19.1 ± 0.203 (n = 6)	18.1 ± 0.267 (n = 6)	5.2 *

Values are means ± SEM; n, number of mice
