# Supplementary material for: Persistence of Burkholderia thailandensis E264 in lung tissue after a single binge alcohol episode
Source: PLoS One. 2019 Dec 10;14(12):e0218147. doi: 10.1371/journal.pone.0218147 (PMC6903738; doi:10.1371/journal.pone.0218147)
Supplement: S3 Table — Mice were administered alcohol (4.4 g/kg) and (0.5, 3, 6, or 24 h) later mice were intranasally infected with B. thailandensis (5 x 10^5^). Mice were weighed before infection and 24 h post infection. PBS (control) indicates mice that were not infected or administered alcohol. Alcohol (control) indicates mice that were administered alcohol and not infected. (*) indicates statistical comparison between pre-infection and post-infection (24 h) per group by Student’s unpaired t-test, *, p ≤ 0.05, **, p ≤ 0.01, ***, p ≤ 0.001. (PDF) [file pone.0218147.s003.pdf]

S3 Table. Average body weight of C57BL/6 mice administered binge-alcohol doses at different times prior to infection. Mice were administered alcohol (4.4 g/kg) and (0.5, 3, 6, or 24 h) later mice were intranasally infected with *B. thailandensis* (5 x 10^5). Mice were weighed before infection and 24 h post infection. PBS (control) indicates mice that were not infected or administered alcohol. Alcohol (control) indicates mice that were administered alcohol and not infected. (*) indicates statistical comparison between pre-infection and post-infection (24 h) per group by Student's unpaired *t*-test, *, p ≤ 0.05, **, p ≤ 0.01, ***, p ≤ 0.001.

	Body Weight (g)		
Alcohol Before Infect.(h)	Pre-Infection	Post-Infection (24 h)	Decrease (%)
PBS (control)	20.1 ± 0.211 (n = 6)	20.4 ± 0.112 (n = 6)	0.0
Alcohol (control)	19.7 ± 0.158 (n = 6)	19.8 ± 0.212 (n = 6)	0.0
0.5	20.4 ± 0.584 (n = 6)	18.5 ± 0.549 (n = 6)	9.3 *
3	19.3 ± 0.407 (n = 6)	17.5 ± 0.538 (n = 6)	9.3 *
6	19.3 ± 0.231 (n = 6)	17.2 ± 0.338 (n = 6)	10.8 ***
24	19.6 ± 0.373 (n = 6)	17.7 ± 0.359 (n = 6)	9.7 **

Values are means ± SEM; n, number of mice
