# Supplementary material for: Persistence of Burkholderia thailandensis E264 in lung tissue after a single binge alcohol episode
Source: PLoS One. 2019 Dec 10;14(12):e0218147. doi: 10.1371/journal.pone.0218147 (PMC6903738; doi:10.1371/journal.pone.0218147)
Supplement: S4 Table — Whole blood cultures were collected 24 h post infection via cardiac puncture and viable B. thailandensis E264 was grown on LB media plates to determine colony forming units (CFUs). B. thailandensis E264 colonies confirmed per plate and assay. Whole blood CFUs represent average number of colonies for each group within a specific experimental assay; 1. Bacterial dosage (inoculum CFUs), 2. Alcohol dosage (administered alcohol), or 3. Temporal effects (alcohol before infection). (PDF) [file pone.0218147.s004.pdf]

S4 Table. Average *B. thailandensis* detected in C57BL/6 whole blood. Whole blood cultures were collected 24 h post infection via cardiac puncture and viable *B. thailandensis* E264 was grown on LB media plates to determine colony forming units (CFUs). *B. thailandensis* E264 colonies confirmed per plate and assay. Whole blood CFUs represent average number of colonies for each group within a specific experimental assay; 1. Bacterial dosage (inoculum CFUs), 2. Alcohol dosage (administered alcohol), or 3. Temporal effects (alcohol before infection).

	Bacteria Detected in Whole Blood (CFUs)
1. Bacterial Dosage: Inoculum (CFUs)	
3×10^5	$3.2 \times 10^3 \pm 0.112$ (n = 6)
5×10^4	$5.2 \times 10^2 \pm 0.211$ (n = 6)
8×10^3	0.0 (n = 6)
500	0.0 (n = 6)
0<br>(PBS & BAC 0.254 %)	0.0 (n = 6)
2. Alcohol Dosage: Administered Alcohol (g/kg)	
4.4	$3.5 \times 10^3 \pm 0.154$ (n = 6)
3	$2.2 \times 10^3 \pm 0.132$ (n = 6)
2	0.0 (n = 6)
1	0.0 (n = 6)
0<br>(3×10^5 CFUs & PBS)	0.0 (n = 6)
3. Temporal Effects: Alcohol Before Infection (h)	
0.5	$3.3 \times 10^3 \pm 0.124$ (n = 6)
3	$1.9 \times 10^3 \pm 0.128$ (n = 6)
6	0.0 (n = 6)
24	0.0 (n = 6)
Control<br>(5×10^5 CFUs & PBS 0.5 h before infection)	0.0 (n = 6)

Values are means ± SEM; n, number of mice
